# Elevation-Related Variation in Leaf Stomatal Traits as a Function of Plant Functional Type: Evidence from Changbai Mountain, China

**DOI:** 10.1371/journal.pone.0115395

**Published:** 2014-12-17

**Authors:** Ruili Wang, Guirui Yu, Nianpeng He, Qiufeng Wang, Fucai Xia, Ning Zhao, Zhiwei Xu, Jianping Ge

**Affiliations:** 1 Synthesis Research Center of Chinese Ecosystem Research Network, Key Laboratory of Ecosystem Network Observation and Modeling, Institute of Geographic Sciences and Natural Resources Research, Chinese Academy of Sciences, Beijing, China; 2 University of Chinese Academy of Sciences, Beijing, China; 3 Forestry College of Beihua University, Jilin, China; 4 College of Life Sciences, Beijing Normal University, Beijing, China; Institute of Botany, China

## Abstract

Understanding the variation in stomatal characteristics in relation to climatic gradients can reveal the adaptation strategies of plants, and help us to predict their responses to future climate changes. In this study, we investigated stomatal density (SD) and stomatal length (SL) in 150 plant species along an elevation gradient (540–2357 m) in Changbai Mountain, China, and explored the patterns and drivers of stomatal characteristics across species and plant functional types (PFTs: trees, shrubs, and herbs). The average values of SD and SL for all species combined were 156 mm^–2^ and 35 µm, respectively. SD was higher in trees (224 mm^–2^) than in shrubs (156 mm^–2^) or herbs (124 mm^–2^), and SL was largest in herbs (37 µm). SD was negatively correlated with SL in all species and PFTs (*P*<0.01). The relationship between stomatal characteristics and elevation differed among PFTs. In trees, SD decreased and SL increased with elevation; in shrubs and herbs, SD initially increased and then decreased. Elevation-related differences in SL were not significant. PFT explained 7.20–17.6% of the total variation in SD and SL; the contributions of CO_2_ partial pressure (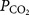), precipitation, and soil water content (SWC) were weak (0.02–2.28%). Our findings suggest that elevation-related patterns of stomatal characteristics in leaves are primarily a function of PFT, and highlight the importance of differences among PFTs in modeling gas exchange in terrestrial ecosystems under global climate change.

## Introduction

Stomata, small pores on the surfaces of plant leaves and stalks, act as turgor-operated valves in controlling the exchange of gases (e.g., water vapor and CO_2_) between plant tissues and the atmosphere [1], [2]. Stomata therefore play a major role in regulation of water and carbon cycling. The morphology, distribution, and behavior of stomata vary in response to environmental changes on timescales from moments to millennia [3]. Stomatal morphology and distribution are controlled primarily by genetic characteristics and phenotypic plasticity, reflecting long-term adaptations of plant species to their growth environment. However, stomatal behaviors (opening and closing) are short-term responses to environmental changes [2], [4]. Therefore, stomatal density (SD) and size, which are relatively stable, are better characteristics for understanding the adaptation or response of plant species to changing environmental conditions at large spatial scales.

Elevation gradients provide a setting for powerful “natural experiments” in which ecological and evolutionary responses of biota to changing environments can be tested [5]. Along elevation gradients, large changes in environmental factors occur over short distances, leading to apparent changes in the selection pressures imposed on plant life-history strategies and traits [4]. To some extent, spatial variation in plant traits along elevation gradients can reflect the trends associated with climate warming [6], [7]. SD has been reported to vary in different ways along environmental gradients, either increasing [8], [9], decreasing [10], or remaining unchanged [11]. Some researchers have observed an initial increase in SD, followed by a decrease, with increasing elevation [12]-[14]. There is a trade-off between SD and size in terms of the exchange of CO_2_ and water [11], [15]. SD is more responsive to environmental conditions than stomatal size [16], [17]. Previous studies showed either decreased or increased in stomatal size with increasing elevation [11], [18].

Various hypotheses have been developed to explain the relationships between stomatal characteristics and elevation. The reduced CO_2_-availability hypothesis suggests that photosynthesis can be impeded at high elevations by the decline in partial pressure of CO_2_ (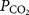), and that plants may increase their SD or stomatal conductance to enhance carbon gain during the short growing season [8], [19]. A second hypothesis, the drought stress theory, proposes that high elevation may affect leaf structure via a drought effect [10], [13]. As elevation increases, the diffusion coefficient of water vapor in air increases, and water uptake by roots decreases because the soil temperature is lower [13], [20], which results in water stress; under these conditions, SD may be reduced as a water conservation mechanism [10], [13]. However, some studies demonstrated that SD increased with elevation to meet the demand for high transpiration [21]. The third theory, proposed by Körner et al. [22], [23], attributes the increase in SD to the increased interception of solar radiation with elevation, where light intensity has a positive effect on SD [24]. These three hypotheses help to explain the mechanisms that underlie changes in SD along elevation gradients. However, changes in rainfall, wind exposure, and other factors may make elevation-related patterns in stomatal characteristics difficult to predict.

Changbai Mountain is a volcanic mountain in northeastern China; the vertical distribution of vegetation on this mountain mirrors the horizontal vegetation types of temperate and frigid zones in Eurasia [25], [26]. Here, we investigated SD and stomatal length (SL) in 150 species along an elevation gradient on Changbai Mountain. The majority of studies of stomatal patterns have focused on one or a few species [8], [13], little is known about general stomatal patterns along altitudinal gradients in a broad, interspecific context. Furthermore, whether altitudinal variation in SD and SL is related to plant functional types (PFTs) has not been addressed. In this study, we aimed to obtain a general understanding of variation in plant stomata along altitudinal gradients at the species or PFT level. Our specific objectives were to assess (1) the elevation-related changes in SD and SL across plant species and PFTs, and (2) the effects of PFT and environmental variables on elevation-related variation in stomatal characteristics.

## Materials and Methods

### Ethics statement

We obtained special permission from Changbai Mountain National Reserve, Jilin Province, China, for our field investigation. We have no commercial interests or conflicts of interest in performing this work. We confirm that this study did not involve endangered or protected species, and no protected species were sampled during the monitoring time.

### Site description and sampling

This study was conducted on the northern slope of Changbai Mountain (41°23′–42°36′N, 126°55′–129°00′E) in Jilin Province, China. This region has a temperate continental climate with long cold winters and warm summers. As elevation increases from 530 to 2200 m, mean annual temperature (MAT) decreases from 2.9 to –4.8°C, and mean annual precipitation (MAP) increases from 632 to 1154 mm [25]. Topographic and climatic variation results in vertical zonation of major forest types along the northern slope of Changbai Mountain. Deciduous broadleaved forest dominated by *Quercus mongolica* is present at elevations below 700 m. From 700 to 1100 m, the typical temperate forest is composed of Korean pine (*Pinus koraiensis*) and hardwood species. Coniferous forest dominated by spruce (*Abies nephrolepis*) and fir spruce (*Picea jezoensis*) is present from 1100 to 1700 m. Erman's birch (*Betula ermanii*) forest dominated by mountain birch and larch (*Larix olgensis*) occurs from 1700 to 2000 m. The southernmost occurrence of alpine tundra in eastern Eurasian occurs above 2000 m, and is dominated by *Dryas octopetala* and *Rhododendron chrysanthum* [25], [27].

Six sampling sites were located on the northern slope of Changbai Mountain along an elevation gradient (Table 1); four experimental plots (30 × 40 m) were established in each site. In each plot, leaf samples were collected according to the protocols of Cornelissen et al. [28]. Briefly, 20 fully expanded sun leaves were collected from four individuals of each plant species. In total, 150 plant species from 105 genera and 47 families were sampled. Some species occurred frequently and some occurred at only one or two sites (Table S1). Soil samples (0–10 cm) were collected from random locations in each plot, and then mixed thoroughly.

**Table 1:** Major characteristics of six sampling sites along an elevation gradient on Changbai Mountain.

Elevation (m a.s.l.)	Latitude	Longitude	Soil type	Forest type	MAP† (mm)	MAT (°C)	SWC (%)	TN (mg g^–1^)	TC (mg g^–1^)
**540**	42°37′	128°4′	Albi-Boric Argosols	Broad-leaved	632	2.9	122.24^a^ ‡	10.45^a^	132.24^a^
**753**	42°24′	128°5′	Albi-Boric Argosols	Mixed coniferous broad-leaved	691	2.6	97.64^b^	7.59^b^	97.06^b^
**1286**	42°8′	128°11′	Bori-Udic Cambisols	Dark-coniferous spruce-fir	811	0.3	35.16^c^	1.22^d^	26.16^c^
**1812**	42°04′	128°04′	Umbri-Gelic Cambisols	Erman's birch	967	–2.3	62.73^b^	5.18^c^	75.71^b^
**2008**	42°03′	128°03′	Permi-Gelic Cambisols	Alpine tundra	1038	–3.3	74.00^b^	3.92^c^	64.58^b^
**2357**	42°02′	128°03′	Permafrost cold Cambisols	Alpine tundra	1154	–4.8	48.14^c^	2.9^d^	42.60^b^

† MAP, mean annual precipitation; MAT, mean annual temperature; TN, soil total nitrogen content; TC, soil total carbon content. MAP and MAT are cited from Shen et al. [25]. Soil samples are collected at 0–10 cm depth.

‡ Means with different lowercase letters differ significantly among elevations (*P*<0.05).

### Stomatal observations

For each species, three leaf individuals were randomly chosen for anatomical study. Stomatal parameters were measured from surface impressions of the mid-blade abaxial leaf surface (avoiding leaf veins) made with clear enamel nail polish [29], [30]. In detail, we first applied clear nail varnish to a 1 cm^2^ patch on the both sides of the main vein on the leaf abaxial surface to make a transparent imprint of the leaf surface. After drying, we removed the nail varnish with pincers, and mounted it on a microscope slide. SD (mm^–2^) was estimated by counting the number of stomata from 3–5 different fields of view on each leaf sample at 400× magnification (for details, see S1 Figure). In each of these images, we measured the guard cell length of three randomly selected stomata to represent SL (µm). Therefore, for each species, we calculated the SD in at least 9 fields of view at 400× magnification (visual field area  =  62,685 µm^2^), and measured the length of approximately 12 guard cells.

All stomatal measurements were conducted with electronic image analysis equipment (COIC XSZ-HS3 and MIPS software, Optical Instrument Co., Ltd., Chongqing, China). We obtained 1138 leaf cuticle images in total.

### Environmental variables

The climatic variables in this study, including MAT and MAP for each sampling site, were derived from Shen et al. [25]. 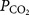
was calculated as [8]: 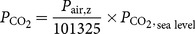
(1)


where 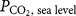 is the partial pressure of CO_2_ at sea level, which is equal to 37.5 Pa; 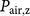
(Pa) is calculated from altitude (z, in meters) and mean July temperature (T, 2°C):
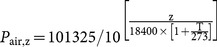
(2)


where T is calculated using a lapse rate of 0.6°C per 100 m of altitude.

Soil water content (SWC, %) was determined after soil was dried at 105°C for 24 h. Then, soil samples were air-dried and sieved, roots were removed by hand, and the samples were ground to pass through a 2-mm mesh. Soil total carbon and total nitrogen (TC and TN, mg g^–1^) were analyzed using an elemental analyzer (Vario MAX CN; Elementar, Germany). Climatic variables (MAT, MAP, and 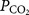) were closely correlated with elevation (S2 Table; *P*<0.01); SWC was not significantly correlated with elevation or other environmental factors (*P*>0.05).

### Data analysis

Species-by-site data (S1 Table) were averaged for each species (species level), and the average values for each species were then classified into trees, shrubs, and herbs (PFT level); categorization by PFT is considered as a convenient means of simplifying diverse plant physiological functions for ecological modeling [31]. Stomatal data were tested for normality and homogeneity of variance, and were log_10_-transformed before analysis when necessary.

Comparisons of SD and SL among altitudes and PFTs were performed with one-way analysis of variance (ANOVA) with least significant difference (LSD) *post hoc* tests. Then, we evaluated the bivariate relationship between SD and SL using standardized major axis estimation (SMA) with the R package “smart” [32]. The program first tested for differences in slope among SMA relationships. If no significant difference in slope was detected (*P*>0.05), tests for differences in elevation (y-intercept) and whether PFTs were separated along the standardized major axis with a common slope were performed using using randomization routines that are analogous to standard analysis of covariance (ANCOVA) [31]. In addition, the relationships between stomatal characteristics and environmental variables and PFT were analyzed with general linear model (GLM) using sequential (type-I) sums of squares. The explanatory terms included PFT, 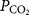, MAP, SWC, and their interactions. Considering the significant correlations among MAT and other climatic variables (Table S2), MAT was excluded from this analysis. Environmental variables that were significantly correlated with stomatal characteristics were further analyzed to quantify their effects on SD and SL using linear regression with ordinary least squares (OLS).

All analyses were conducted with R 2.15.2 [33]. Linear regression and one-way ANOVA with LSD tests were analyzed with *P*<0.05 considered statistically significant.

## Results

### General statistics for SD and SL

**Figure 1:**
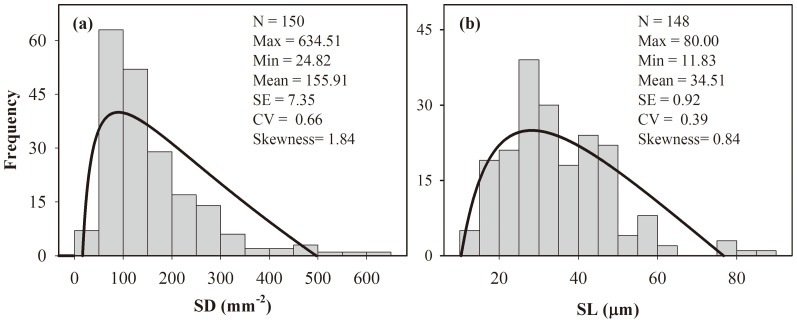
Histograms of the distribution of stomatal density (SD, a) and stomatal length (SL, b) for all species. The fitted log-normal curves are indicated. N, species number; Max, maximum value; Min, minimum value; SE, standard error; CV, coefficient of covariance.

The mean values and ranges of SD and SL for the 150 observed plant species are presented in Fig. 1; both SD and SL were positively skewed, and the variation in SD was larger than that in SL. Stomatal characteristics differed significantly among PFTs (Table 2, *F*  =  19.63, *P*<0.001 for SD; *F*  =  7.82, *P*<0.001 for SL). SD was highest in trees and lowest in herbs (*P*<0.05). In contrast, SL was greatest in herbs, intermediate in trees, and lowest in shrubs.

**Table 2:** Statistics for stomatal density (SD) and stomatal length (SL) of different plant functional types (PFTs) on Changbai Mountain.

Traits	PFT	*n* †	Maximum	Minimum	Mean	SE	CV
SD (mm^-2^)	All species	150	634.51	24.82	155.91	7.53	0.66
	Tree	33	634.51	89.20	224.02^a^ ‡	19.04	0.61
	Shrub	26	347.70	61.15	155.67^b^	15.00	0.56
	Herb	91	322.98	24.82	124.36^b^	6.58	0.56
**SL (µm)**	All species	148	80.00	11.83	34.51	0.92	0.39
	Tree	33	77.48	13.67	33.16^ab^	1.95	0.43
	Shrub	26	42.63	12.67	27.79^a^	1.20	0.25
	Herb	89	80.00	11.83	37.23^b^	1.24	0.35

† *n*, number of species; SE, standard error; CV, coefficient of variation.

‡ Means with different lowercase letters differ significantly among plant functional types (*P*<0.05).

**Figure 2:**
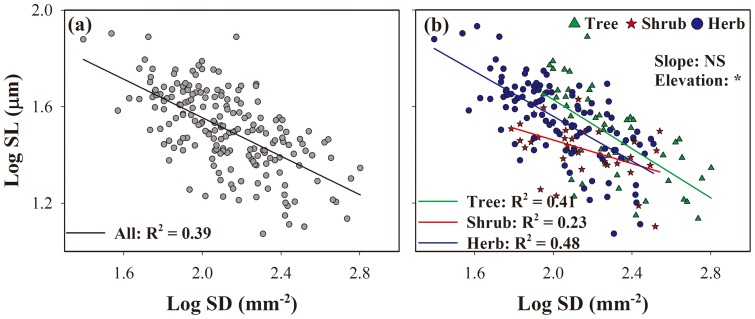
Relationship between stomatal density (SD) and stomatal length (SL) at the species (a) and plant functional type (PFT, b) level. “Slope”, difference in standardized major axis (SMA) slopes; “Elevation”, difference in SMA elevations (y-intercept); NS, not significantly different; *, significantly different (*P*<0.05). All regression lines shown in each panel are significant (*P*<0.01).

Strong negative relationships between SD and SL were observed at the species (Fig. 2a, R^2^  =  0.39, *P* <0.0001) and PFT levels (Fig. 2b, R^2^  =  0.41, *P*  =  0.004 for trees;R^2^  =  0.23, *P* <0.0001 for shrubs; R^2^  =  0.48, *P* <0.0001 for herbs). The slope of the relationship between SD and SL differed (but not significantly) among growth forms (test for SMA heterogeneity, *P*  =  0.07); rather, the elevation (y-intercept) was clearly lower for shrubs (*P*<0.0001).

### Differences in stomatal variation along the elevation gradient

**Figure 3:**
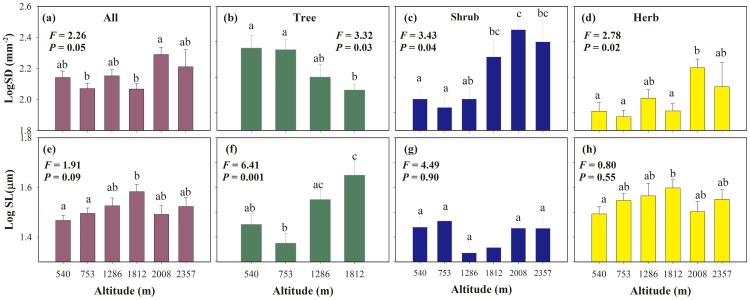
Changes in stomatal density (SD) and stomatal length (SL) with elevation for all species (a, e), trees (b, f), shrubs (c, g), and herbs (d, h). Data are means; bars represent standard error. Different lowercase letters in each panel indicate significant differences among altitudes (*P*<0.05).

No clear trends in SD and SL were observed in relation to elevation for all plant species (Fig. 3a, *F*  =  2.26, *P*  =  0.05 for SD; Fig. 3e, *F*  =  1.91, *P*  =  0.09 for SL). Elevation-related differences in stomatal characteristics varied among PFTs. In trees, SD decreased and SL increased significantly with altitude (Fig. 3b, *F*  =  3.32, *P*  =  0.03 for SD; Fig. 3f, *F*  =  6.41, *P*  =  0.0001 for SL). In shrubs and herbs, the relationships between SD and altitude were nonlinear; SD initially increased, reached a maximum at 2008 m, and then decreased (Fig. 3c, *F*  =  3.43, *P*  =  0.04 for shrubs; Fig. 3d, *F*  =  2.78, *P*  =  0.02 for herbs). There were no significant trends in SL according to altitude (Fig. 3g, *F*  =  4.49, *P*  =  0.90 for shrubs; Fig. 3h, *F*  =  0.80, *P*  =  0.55 for herbs).

### Stomatal variation in relation to PFT, meteorological variables, and soil

GLM analysis revealed that PFT, climatic variables, and SWC jointly explained 28.88% and 18.67% of the variation in SD and SL, respectively (Table 3). Among the environmental factors, only 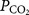 and SWC were significantly correlated with SD or SL (*P*<0.05). PFT was the dominant factor explaining the variation in stomatal characteristics; the joint effect of PFT and 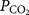 also contributed significantly to the total variation (*P*<0.05), although the independent effects of climatic and soil variables were weak.

**Table 3:** Summary of general linear models (GLM) of stomatal density (SD) and stomatal length (SL).

Factor	Log SD			Log SL		
	DF‡	MS	SS%	DF	MS	SS%
**PFT** †	2	1.16^**^	17.62	2	0.19^**^	7.20
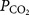	1	0.24*	1.85	1	0.11*	2.01
**Log MAP**	1	0.00	0.02	1	0.00	0.04
**SWC**	1	0.30*	2.28	1	0.03	0.64
**PFT ×** 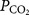	2	0.43^**^	6.47	2	0.14^**^	5.21
**PFT × LogMAP**	2	0.02	0.30	2	0.07	2.58
**PFT × SWC**	2	0.02	0.35	2	0.03	0.99
**Residual**	186	0.05	71.12	184	0.02	81.33

† PFT, plant functional type; 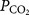, CO_2_ partial pressure; MAP, mean annual precipitation; SWC, soil water content;

‡ DF, degrees of freedom; MS, mean squares; SS%, percentage of sum of squares explained.

*, *P*<0.05; **, *P*<0.01.

**Figure 4:**
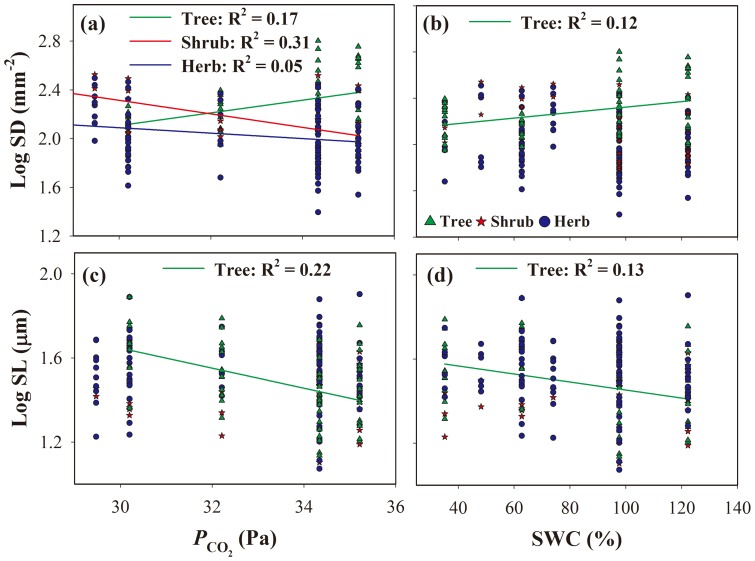
Stomatal density (SD) and stomatal length (SL) in relation to CO_2_ partial pressure (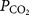; a, c), and soil water content (SWC; b, d). Only significant regression lines are shown in each panel (*P*<0.05).

SD was positively related to 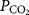
and SWC in trees, and negatively related to these variables in SL (Fig. 4). In shrubs and herbs, SD decreased with increasing 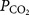
(Fig. 4a), and SL was not correlated with changes in 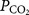
or SWC (Fig. 4c–d).

## Discussion

### Altitudinal patterns in SD and SL in relation to PFT

The ways in which stomatal morphological traits vary with environmental gradients are not fully understood. Some authors have found that a linear increase in SD along environmental gradients in many species [8], [9], [19]. However, other studies have reported fewer stomata at high elevations [10], and no changes [11], or non-linear variation in SD with elevation [12]-[14]. For example, Qiang et al. [12] pointed out that SD of *P. crassifolia* increased significantly with elevation below 3000 m, and then decreased. Körner et al. [23] reported that SD increased with elevation in trees, shrubs, and herbs, and attributed this variability to changes in light availability in the southern Alps of New Zealand.

Here, we investigated the relationships between SD and SL and elevation to better understand the ways in which plants adjust to environmental changes at the species and PFT levels. Our results suggested that no clear trends in SD and SL occurred at the species level, but that altitudinal trends depended on PFT (Fig. 3). In trees, SD decreased and SL increased significantly with increasing elevation, while the relationships between SD and altitude were nonlinear in shrubs and herbs. The discrepancies between our observations and those of Körner et al. [23] probably were a result of differences in the underlying ecological mechanisms, as discussed below.

### Ecological mechanisms behind altitudinal patterns in stomatal variation

Many studies have shown a significant influence of elevation on stomatal characteristics [18], [34]. The elevation effect is a proxy for environmental factors including 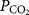, air temperature, solar irradiance, precipitation, and wind exposure [5]. Variation in these combined factors along an elevation gradient could obscure the effects of individual parameters, resulting in a lack of clear patterns of SD in relation to elevation [8], [10], [14], [23]. In this study, elevation-related differences in stomatal characteristics were strongly related to PFT, although no clear trends in SD and SL were observed at the species level. The relationships between leaf morphological and chemical characteristics and PFT have been documented in previous studies [35]-[38]. Reich et al. [35] found that PFT accounted for 33–67% of spatial variation in specific leaf area, photosynthetic rate, and leaf nitrogen and phosphorus content in more than 2000 species sampled from around the world, while climate metrics explained only 5–20% of the total variation. Kelly & Beerling [39] pointed out that SD differed among trees, shrubs, and herbs. However, little is known about the extent to which PFT can explain the variation in SD and SL. Here, we further confirmed that PFT was the main explanatory factor behind elevation-related differences in stomata.

In shrubs and herbs, SD increased and then decreased with increasing elevation, and this variation in SD was significantly affected by 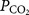
(Fig. 4a), which could be partially explained by the CO_2_-availability theory. The stomatal response to changes in CO_2_ concentration, and the developmental pathway involved in this response has been discussed extensively [15], [30], [40], [41]. The *Arabidopsis* gene *HIC* (for high CO_2_ concentration), which encodes a putative 3-keto acyl coenzyme A synthase, has been shown to negatively regulate stomatal development in response to CO_2_ [42]. Moreover, leaf internal CO_2_ concentration is strongly correlated with the development of stomatal and pavement cells [40], [43]. Thus, when grown under conditions of rising CO_2_, the majority of species respond by reducing SD on the leaf surface [2], [44], and this relationship extends over geological time [15]. Although the CO_2_ mixing ratio in air remains constant over altitudinal gradients, 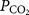
is lower at higher elevations because of the lower air pressure [8]. Some studies have demonstrated that plants grown under lower CO_2_ availability had significant increase in SD to enhance photosynthesis rates [8], [45], which is consistent with the altitudinal trends in SD observed for shrubs and herbs at elevations below 2008 m on Changbai Mountain.

In addition to CO_2_ availability, light intensity has a significant effect on SD by inducing changes in epidermal cell expansion [23], [24]. At lower elevations, shrubs and herbs tend to occur in the understory where competition for sunlight is strong, while at higher elevations, these plants commonly grow in more open habitats [23]. Thus, the increase in SD of shrubs and herbs at elevations below 2008 m might be influenced by changes in light intensity. The lower SD above 2008 m might have been due to severe environmental conditions (e.g., lower temperature, higher UV-B levels and wind velocity) that could inhibit formation of stomata [8], [12].

Contrary to the CO_2_-availability hypothesis, we observed a positive relationship between SD and 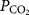
in trees (Fig. 4a), probably because the effects of other environmental factors obscured the effects of atmospheric CO_2_ concentration on SD. Other studies have also suggested that the negative relationship between SD and CO_2_ was not apparent across all species and locations [8], [30], [46]. Yang et al. [18] found that
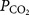
had a positive effect on SD, due to the low temperature and strong insolation in Chinese grassland. Besides 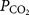, changes in soil moisture condition had a significant effect on the variation in SD and SL of trees in this study (Fig. 4b and Fig. 4d). Thus, stomatal variation in trees may be explained partly by drought stress theory. At high elevations, woody plants are vulnerable to water-deficit stress because low air and soil temperatures reduce water uptake by roots [13], [20]; trees at higher elevations can thus show characteristics of drought acclimation [47], which is consistent with mechanisms of water conservation evolved under drought stress. Relatively lower SD in these species minimizes water loss by leaf transpiration, and increases water use efficiency [48]. In contrast, plants growing in optimum habitats may utilize water extravagantly [13]. Schoettle et al. [10] demonstrated that SD in *P. flexilis* decreased with elevation; minimizing water loss may be more advantageous than increasing CO_2_ uptake in dry, high-elevation habitats. Our observation of reduced SD in trees at higher elevations was consistent with the idea of minimizing water loss under dry conditions.

Changes in woody species composition with elevation can also contribute to the stomatal variation in trees. Specifically, deciduous woody angiosperms dominated at lower altitudes on Changbai Mountain, while species at higher altitudes primarily belonged to coniferous gymnosperms (S1 Table and S3 Table). Previous studies have suggested that the angiosperms are characterized with many small stomata to reach high stomatal conductance, and the conifers cluster with few large stomata [49]. In order to eliminate genetic differences as a factor, and to test whether individual species show the same response as functional groups to elevation, we further analyzed the altitudinal variation in SD and SL in *P. koraiensis* (S2 Figure). *P. koraiensis* was chosen since it was the only species that occurred in all vegetation zones along the elevation gradient. Our results suggested that SD reached a maximum, and SL a minimum, at 753 m. Although this pattern differed somewhat from the altitudinal variation in SD of trees, SD of *P. koraiensis* was still found to decrease along the elevation gradient above 753 m.

### Trade-off between SD and SL

Stomatal control determines the balance of CO_2_ uptake needed for plant photosynthesis against water loss by transpiration [2]. In the short term, plants modulate the width of stomatal aperture in response to environmental changes. In the long term, plants are able to adapt stomatal development and morphology to their growth environment through natural selection. In the present study, SL was negatively correlated with SD across species and growth forms (Fig. 2). This is consistent with the strong and pervasive trade-off between SD and SL across multiple species and geological time-scales reported by other studies [2], [15]. The coordination of the size and number of stomata is thought to maximize carbon gain, while minimizing water loss under fluctuating environmental conditions [2], [15].

Smaller stomata can respond quickly to environmental changes by opening and closing rapidly; and their association with high densities of stomata enables rapid increases in stomatal conductance to maximize CO_2_ diffusion when conditions for photosynthesis are favorable [2]. Although stomatal size has strong ecological significance, little attention has been paid to elevation-related changes in stomatal size [11]. One explanation is that stomatal size shows strong phylogenetic conservation (i.e. closely related species have similar responses to environmental changes), and thus lower plasticity than SD in relation to environmental conditions [17].

## Conclusions

Altitudinal trends in SD and SL differed significantly among PFTs, and there was a strong negative correlation between SD and SL at the species and PFT levels. In trees, SD decreased and SL increased with increasing elevation, while SD showed non-linear relationships to elevation in shrubs and herbs, although no clear patterns were observed among all species. Altitudinal variation in SD and SL was mainly influenced by PFT, but was also related to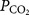, SWC,and the joint effects of PFT and 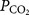. It is difficult to generalize about species-level variation in plant stomata along altitudinal gradients, and differences in stomatal characteristics with elevation are mainly a function of PFT. These findings provide new insights for exploring plant adaptations or responses to changing environmental conditions at large spatial scales.

## Supporting Information

S1 Figure
**Detailed method to calculate stomatal density (SD) and stomal length (SL).** Take the leaf image of *Gentiana algida* at 400× magnification for example. In this picture, SD is calculated as: 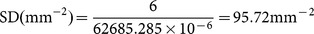

where the stomatal number is 6, and the area of visual field is 62685.285 µm^2^. SL is represented as the guard-cell length; here the value of SL is 56.129 µm. Sale bar  =  20 µm.(TIF)

S2 Figure **Altitudinal variation in stomatal density (SD, a) and stomatal length (SL, b) of** ***P. koraiensis*** **.** Different lowercase letters indicate significant differences (*P*<0.05).
(TIF)

S1 Table **Original data of stomatal density (SD, mm^-2^) and length (SL, µm) at species-by-site level.** GF: growth form; D: deciduous; E: evergreen; N: needle; B: broadleaf.
(docx)

S2 Table
**Correlation matrix for environmental variables.** MAT  =  mean annual temperature, MAP  =  mean annual precipitation, SWC  =  soil water content, and 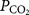 =  CO_2_ partial pressure. Pearson coefficients in bold and with asterisks indicate the correlation is significant at *P*<0.01.(docx)

S3 Table **Number of sampled species among plant functional types (PFTs) at each site along altitude on the Changbai Mountain, China.** The percentage of species number in total amount at each site is given between parentheses (%). Notably, most of evergreen trees are coniferous gymnosperms.
(docx)
