# Supplementary figures and images for: Elevation-Related Variation in Leaf Stomatal Traits as a Function of Plant Functional Type: Evidence from Changbai Mountain, China
Source: PLoS One. 2014 Dec 17;9(12):e115395. doi: 10.1371/journal.pone.0115395 (PMC4269444; doi:10.1371/journal.pone.0115395)

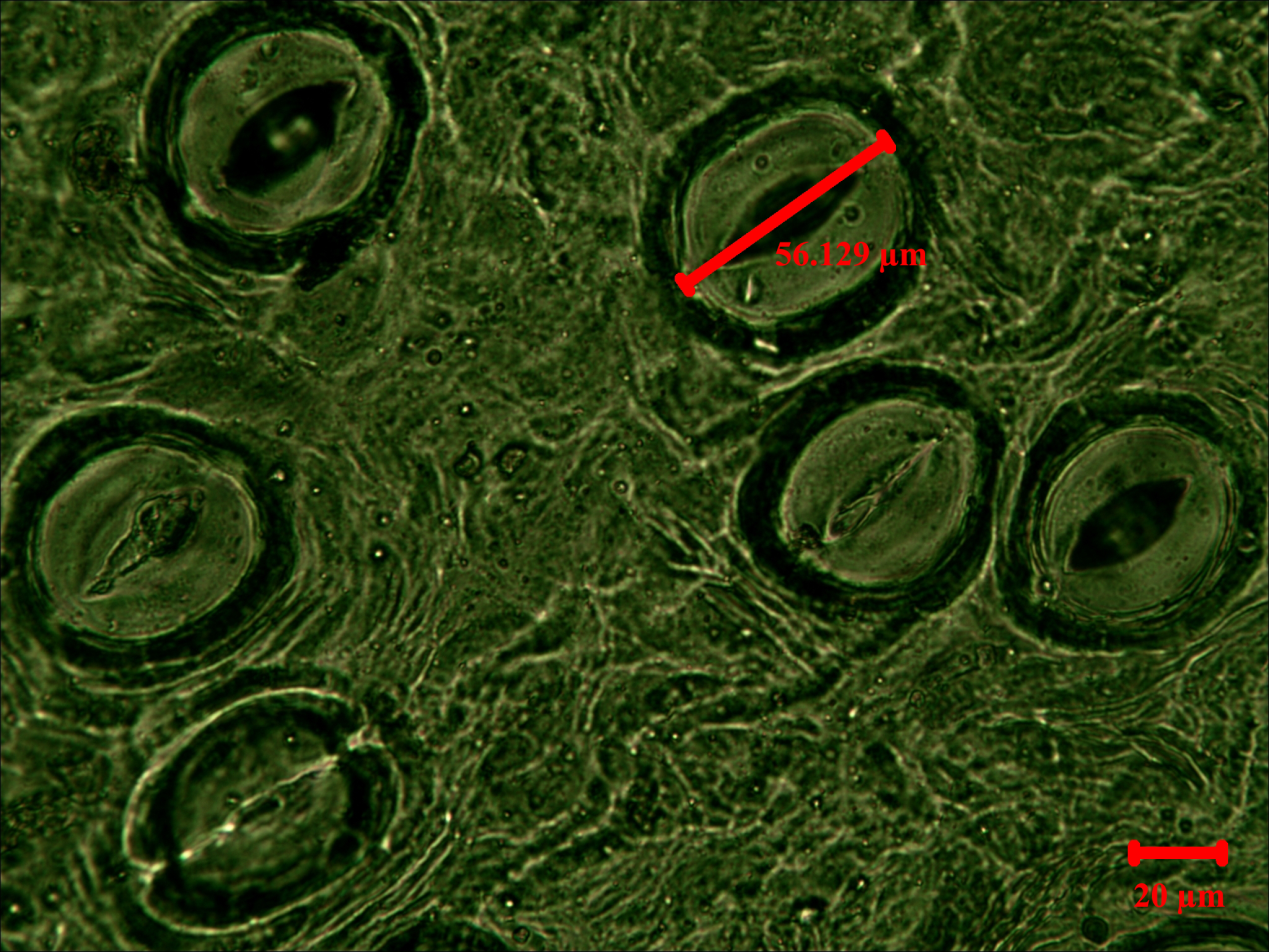

Supplement: S1 Figure — Detailed method to calculate stomatal density (SD) and stomal length (SL). Take the leaf image of Gentiana algida at 400× magnification for example. In this picture, SD is calculated as: where the stomatal number is 6, and the area of visual field is 62685.285 µm2. SL is represented as the guard-cell length; here the value of SL is 56.129 µm. Sale bar = 20 µm. (TIF) [file pone.0115395.s001.tif]

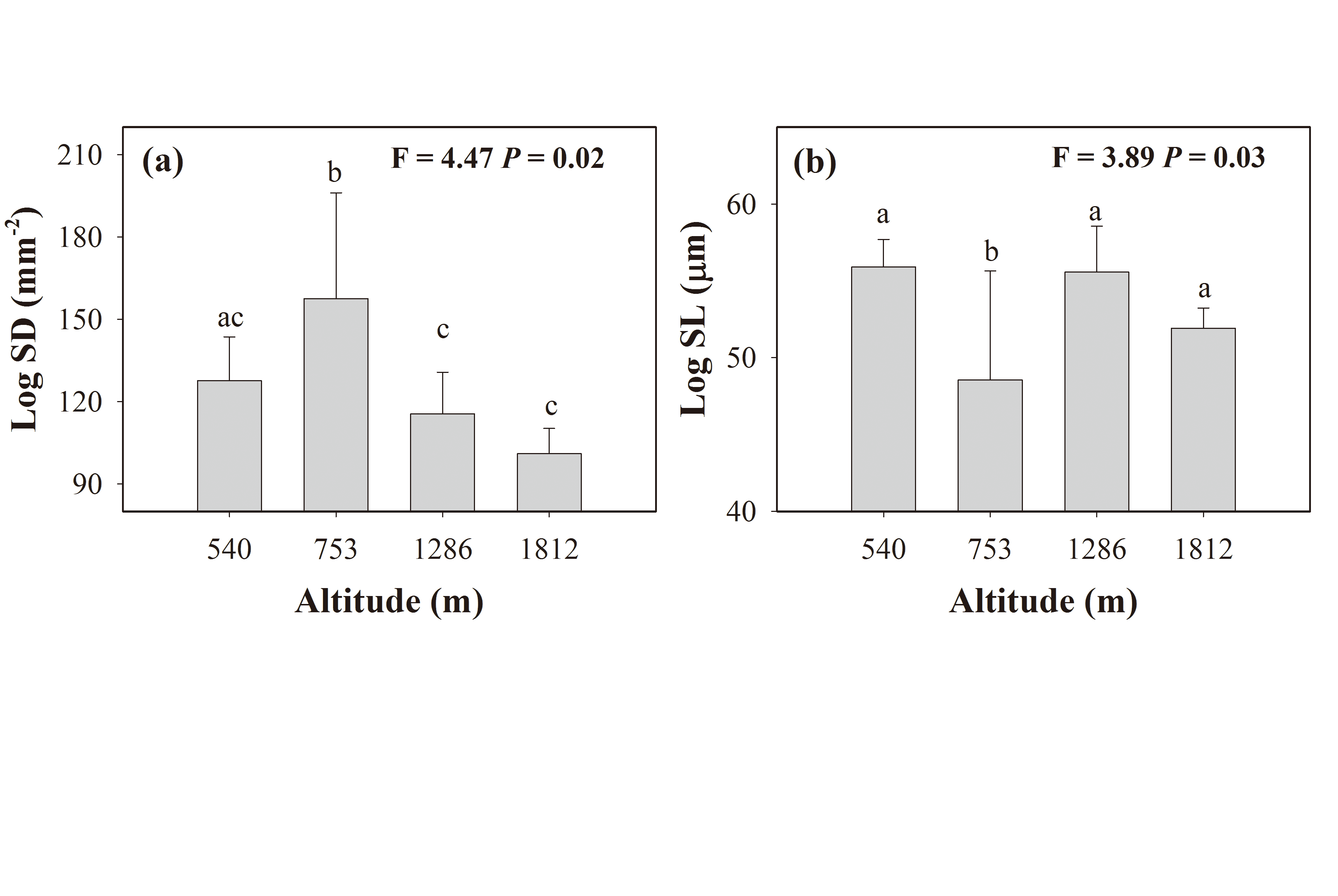

Supplement: S2 Figure — Altitudinal variation in stomatal density (SD, a) and stomatal length (SL, b) of P. koraiensis . Different lowercase letters indicate significant differences (P<0.05). (TIF) [file pone.0115395.s002.tif]
