# Supplementary material for: Elevation-Related Variation in Leaf Stomatal Traits as a Function of Plant Functional Type: Evidence from Changbai Mountain, China
Source: PLoS One. 2014 Dec 17;9(12):e115395. doi: 10.1371/journal.pone.0115395 (PMC4269444; doi:10.1371/journal.pone.0115395)
Supplement: S1 Table — Original data of stomatal density (SD, mm-2) and length (SL, µm) at species-by-site level. GF: growth form; D: deciduous; E: evergreen; N: needle; B: broadleaf. (docx) [file pone.0115395.s003.docx]

**Table S1.** **Original data of stomatal density (SD, mm^-2^) and length (SL, μm) at species-by-site level.** GF: growth form; D: deciduous; E: evergreen; N: needle; B: broadleaf.

| No. | Altitude (m) | Species | GF | D/E | N/B | SD | SL |
| --- | --- | --- | --- | --- | --- | --- | --- |
| 1 | 540 | *Acer pictum* | Tree | D | B | 384.79 | 19.56 |
| 2 | 540 | *Acer triflorum* | Tree | D | B | 460.55 | 16.39 |
| 3 | 540 | *Adenocaulon himalaicum* | Herb |  |  | 127.62 | 25.97 |
| 4 | 540 | *Adiantum pedatum* | Herb |  |  | 54.24 | 31.87 |
| 5 | 540 | *Aegopodium alpestre* | Herb |  |  | 87.74 | 36.77 |
| 6 | 540 | *Angelica anomala* | Herb |  |  | 194.62 | 31.83 |
| 7 | 540 | *Artemisia keiskeana* | Herb |  |  | 91.73 | 27.5 |
| 8 | 540 | *Athyrium multidentatum* | Herb |  |  | 70.19 | 46.85 |
| 9 | 540 | *Athyrium sinense* | Herb |  |  | 57.43 | 39.55 |
| 10 | 540 | *Campanula punctata* | Herb |  |  | 191.43 | 19.15 |
| 11 | 540 | *Carex pilosa* | Herb |  |  | 99.7 | 34.3 |
| 12 | 540 | *Cicuta virosa* | Herb |  |  | 183.46 | 26.66 |
| 13 | 540 | *Cimicifuga dahurica* | Herb |  |  | 124.43 | 32.74 |
| 14 | 540 | *Crataegus pinnatifida* | Shrub | D | B | 134 | 42.63 |
| 15 | 540 | *Eleutherococcus senticosus* | Shrub | D | B | 67.8 | 28.76 |
| 16 | 540 | *Euonymus alatus* | Shrub | D | B | 98.91 | 33.88 |
| 17 | 540 | *Euphorbia pekinensis* | Herb |  |  | 114.33 | 22.66 |
| 18 | 540 | *Fraxinus mandschurica* | Tree | D | B | 417.88 | 28.32 |
| 19 | 540 | *Juglans mandshurica* | Tree | D | B | 246.33 | 35.89 |
| 20 | 540 | *Lamium barbatum* | Herb |  |  | 127.62 | 26.71 |
| 21 | 540 | *Lilium distichum* | Herb |  |  | 34.56 | 80 |
| 22 | 540 | *Lonicera maackii* | Shrub | D | B | 188.24 | 22.78 |
| 23 | 540 | *Maackia amurensis* | Tree | D | B | 140.36 | 18.93 |
| 24 | 540 | *Maianthemum japonicum* | Herb |  |  | 63.81 | 27.81 |
| 25 | 540 | *Malus baccata* | Tree | D | B | 171.46 | 36.54 |
| 26 | 540 | *Padus avium* | Tree | D | B | 478.58 | 16.05 |
| 27 | 540 | *Padus maackii* | Tree | D | B | 138.26 | 26.19 |
| 28 | 540 | *Phellodendron amurense* | Tree | D | B | 103.67 | 43.36 |
| 29 | 540 | *Philadelphus schrenkii* | Shrub | D | B | 77.1 | 28.34 |
| 30 | 540 | *Picea koraiensis* | Tree | E | N | 105.29 | 46.46 |
| 31 | 540 | *Pimpinella brachycarpa* | Herb |  |  | 127.62 | 26.51 |
| 32 | 540 | *Pinus koraiensis* | Tree | E | N | 127.62 | 55.9 |
| 33 | 540 | *Plagiorhegma dubium* | Herb |  |  | 73.38 | 35.67 |
| 34 | 540 | *Populus davidiana* | Tree | D | B | 255.2 | 35.02 |
| 35 | 540 | *Prenanthes tatarinowii* | Herb |  |  | 79.76 | 26.25 |
| 36 | 540 | *Prinsepia sinensis* | Shrub | D | B | 89.34 | 39.35 |
| 37 | 540 | *Pyrola asarifolia* | Herb |  |  | 255.24 | 29.39 |
| 38 | 540 | *Quercus mongolica* | Tree | D | B | 569.4 | 20.05 |
| 39 | 540 | *Rhamnus parvifolia* | Shrub | D | B | 247.27 | 24.52 |
| 40 | 540 | *Ribes komarovii* | Shrub | D | B | 134 | 25.65 |
| 41 | 540 | *Ribes mandshuricum* | Shrub | D | B | 86.14 | 18.05 |
| 42 | 540 | *Sanicula rubriflora* | Herb |  |  | 127.62 | 26.12 |
| 43 | 540 | *Spiraea chamaedryfolia* | Shrub | D | B | 271.2 | 15.45 |
| 44 | 540 | *Tilia amurensis* | Tree | D | B | 145.32 | 31.42 |
| 45 | 540 | *Ulmus japonica* | Tree | D | B | 451.15 | 24.43 |
| 46 | 540 | *Viburnum burejaeticum* | Shrub | D | B | 79.76 | 37.18 |
| 47 | 753 | *Acer barbinerve* | Tree | D | B | 123.63 | 19.74 |
| 48 | 753 | *Acer mandshuricum* | Tree | D | B | 295.13 | 16.09 |
| 49 | 753 | *Acer pictum* | Tree | D | B | 215.36 | 18.2 |
| 50 | 753 | *Acer pseudosieboldianum* | Tree | D | B | 263.22 | 14.1 |
| 51 | 753 | *Acer triflorum* | Tree | D | B | 544.67 | 13.67 |
| 52 | 753 | *Aconitum coreanum* | Herb |  |  | 51.05 | 62.31 |
| 53 | 753 | *Aconitum kusnezoffii* | Herb |  |  | 57.43 | 46.12 |
| 54 | 753 | *Actinidia arguta* | Shrub | D | B | 83.75 | 29.57 |
| 55 | 753 | *Adenophora divaricata* | Herb |  |  | 79.76 | 36.82 |
| 56 | 753 | *Adiantum pedatum* | Herb |  |  | 67.8 | 23.83 |
| 57 | 753 | *Aegopodium alpestre* | Herb |  |  | 124.43 | 33.27 |
| 58 | 753 | *Angelica cartilaginomarginata* | Herb |  |  | 143.57 | 37.45 |
| 59 | 753 | *Artemisia stolonifera* | Herb |  |  | 107.68 | 30.59 |
| 60 | 753 | *Aruncus sylvester* | Herb |  |  | 276.51 | 12.95 |
| 61 | 753 | *Asyneuma japonicum* | Herb |  |  | 115.66 | 34.15 |
| 62 | 753 | *Athyrium multidentatum* | Herb |  |  | 63.81 | 48.08 |
| 63 | 753 | *Athyrium niponicum* | Herb |  |  | 187.96 | 49.42 |
| 64 | 753 | *Brachybotrys paridiformis* | Herb |  |  | 121.24 | 21.15 |
| 65 | 753 | *Caltha palustris* | Herb |  |  | 55.83 | 57.06 |
| 66 | 753 | *Cardamine leucantha* | Herb |  |  | 203.4 | 11.83 |
| 67 | 753 | *Carex bostrychostigma* | Herb |  |  | 42.54 | 43.12 |
| 68 | 753 | *Carex forficula* | Herb |  |  | 37.22 | 38.33 |
| 69 | 753 | *Carex pilosa* | Herb |  |  | 124.96 | 45.61 |
| 70 | 753 | *Caulophyllum robustum* | Tree | E | N | 157.53 | 48.54 |
| 71 | 753 | *Caulophyllum robustum* | Herb |  |  | 69.79 | 43.64 |
| 72 | 753 | *Chrysosplenium lectus-cochleae* | Herb |  |  | 191.43 | 41.75 |
| 73 | 753 | *Corylus mandshurica* | Shrub | D | B | 61.15 | 32.19 |
| 74 | 753 | *Deutzia parviflora var. amurensis* | Shrub | D | B | 67.8 | 33.72 |
| 75 | 753 | *Doellingeria scabra* | Herb |  |  | 82.04 | 41.41 |
| 76 | 753 | *Dryopteris crassirhizoma* | Herb |  |  | 85.75 | 48.65 |
| 77 | 753 | *Eleutherococcus senticosus* | Shrub | D | B | 107.68 | 30.98 |
| 78 | 753 | *Eleutherococcus sessiliflorus* | Shrub | D | B | 71.79 | 26.88 |
| 79 | 753 | *Enemion raddeanum* | Herb |  |  | 115.66 | 26.19 |
| 80 | 753 | *Euonymus alatus* | Shrub | D | B | 143.57 | 31.21 |
| 81 | 753 | *Euonymus phellomanus* | Shrub | D | B | 114.33 | 29.36 |
| 82 | 753 | *Filipendula intermedia* | Herb |  |  | 137.19 | 31.01 |
| 83 | 753 | *Filipendula palmata* | Herb |  |  | 178.14 | 24.08 |
| 84 | 753 | *Fraxinus mandschurica* | Tree | D | B | 361.59 | 25.03 |
| 85 | 753 | *Galium aparine* | Herb |  |  | 79.76 | 45.99 |
| 86 | 753 | *Isodon excisus* | Herb |  |  | 263.22 | 18.79 |
| 87 | 753 | *Isodon japonicus* | Herb |  |  | 264.81 | 15.91 |
| 88 | 753 | *Juglans mandshurica* | Tree | D | B | 181.86 | 33.48 |
| 89 | 753 | *Lychnis cognata* | Herb |  |  | 61.15 | 44.83 |
| 90 | 753 | *Maackia amurensis* | Tree | D | B | 127.62 | 16.88 |
| 91 | 753 | *Maianthemum japonicum* | Herb |  |  | 47.86 | 57.25 |
| 92 | 753 | *Meehania urticifolia* | Herb |  |  | 203.4 | 26.24 |
| 93 | 753 | *Onoclea sensibilis* | Herb |  |  | 92.53 | 45.5 |
| 94 | 753 | *Ostericum grosseserratum* | Herb |  |  | 91.73 | 34.72 |
| 95 | 753 | *Oxalis corniculata* | Herb |  |  | 172.29 | 24.95 |
| 96 | 753 | *Parasenecio hastatus* | Herb |  |  | 87.74 | 42.49 |
| 97 | 753 | *Phellodendron amurense* | Tree | D | B | 116.81 | 40.04 |
| 98 | 753 | *Philadelphus schrenkii* | Shrub | D | B | 115.66 | 31.72 |
| 99 | 753 | *Pinus koraiensis* | Tree | E | N | 164.09 | 48.54 |
| 100 | 753 | *Polemonium caeruleum* | Herb |  |  | 85.08 | 41.87 |
| 101 | 753 | *Polygonatum odoratum* | Herb |  |  | 54.24 | 50.31 |
| 102 | 753 | *Populus cathayana* | Tree | D | B | 207.39 | 25.62 |
| 103 | 753 | *Populus davidiana* | Tree | D | B | 233.97 | 23.9 |
| 104 | 753 | *Pteridium aquilinum* | Herb |  |  | 54.69 | 52.73 |
| 105 | 753 | *Quercus mongolica* | Tree | D | B | 634.51 | 22.18 |
| 106 | 753 | *Ribes mandshuricum* | Shrub | D | B | 124.43 | 29.2 |
| 107 | 753 | *Sanicula rubriflora* | Herb |  |  | 153.15 | 16.31 |
| 108 | 753 | *Saussurea neoserrata* | Herb |  |  | 55.83 | 43.63 |
| 109 | 753 | *Saussurea tenerifolia* | Herb |  |  | 63.81 | 38.83 |
| 110 | 753 | *Sorbaria sorbifolia* | Shrub | D | B | 170.16 | 31.25 |
| 111 | 753 | *Spiraea pubescens* | Shrub | D | B | 329.69 | 12.67 |
| 112 | 753 | *Synurus deltoides* | Herb |  |  | 76.57 | 40.61 |
| 113 | 753 | *Tilia amurensis* | Tree | D | B | 180.04 | 31.8 |
| 114 | 753 | *Tilia mandshurica* | Tree | D | B | 118.05 | 26.49 |
| 115 | 753 | *Trillium kamtschaticum* | Herb |  |  | 24.82 | 75.64 |
| 116 | 753 | *Trollius japonicus* | Herb |  |  | 72.93 | 56.66 |
| 117 | 753 | *Ulmus davidiana var. japonica* | Tree | D | B | 437.1 | 29.12 |
| 118 | 753 | *Urtica angustifolia* | Herb |  |  | 143.57 | 18.21 |
| 119 | 753 | *Viburnum burejaeticum* | Shrub | D | B | 63.81 | 42.31 |
| 120 | 1286 | *Abies fabri* | Tree | E | N | 119.47 | 46.69 |
| 121 | 1286 | *Abies nephrolepis* | Tree | E | N | 150.19 | 42.54 |
| 122 | 1286 | *Alnus hirsuta* | Tree | D | B | 191.15 | 24.92 |
| 123 | 1286 | *Betula platyphylla* | Tree | D | B | 236.66 | 34.98 |
| 124 | 1286 | *Geranium platyanthum* | Herb |  |  | 207.39 | 26.26 |
| 125 | 1286 | *Gymnocarpium jessoense* | Herb |  |  | 47.86 | 42.78 |
| 126 | 1286 | *Larix gmelinii* | Tree | D | N | 99.56 | 61.39 |
| 127 | 1286 | *Lonicera caerulea* | Shrub | D | B | 139.59 | 21.85 |
| 128 | 1286 | *Malus baccata* | Tree | D | B | 199.11 | 32.32 |
| 129 | 1286 | *Osmunda cinnamomea* | Herb |  |  | 89.34 | 55.94 |
| 130 | 1286 | *Picea jezoensis* | Tree | E | N | 89.2 | 44.76 |
| 131 | 1286 | *Pinus koraiensis* | Tree | E | N | 122.12 | 55.57 |
| 132 | 1286 | *Populus cathayana* | Tree | D | B | 228.66 | 28.14 |
| 133 | 1286 | *Populus davidiana* | Tree | D | B | 211.06 | 20.66 |
| 134 | 1286 | *Populus koreana* | Tree | D | B | 248.49 | 32.46 |
| 135 | 1286 | *Pteridium aquilinum* | Herb |  |  | 156.87 | 41.03 |
| 136 | 1286 | *Pterocarya stenoptera* | Tree | D | B | 175.22 | 26.44 |
| 137 | 1286 | *Ribes triste* | Shrub | D | B | 118.05 | 27.51 |
| 138 | 1286 | *Ribes komarovii* | Shrub | D | B | 103.69 | 16.96 |
| 139 | 1286 | *Saposhnikovia divaricat* | Herb |  |  | 233.97 | 28.83 |
| 140 | 1286 | *Solidago virgaurea* | Herb |  |  | 95.72 | 33.43 |
| 141 | 1286 | *Sorbus pohuashanensis* | Tree | D | B | 114.16 | 34.71 |
| 142 | 1812 | *Abies holophylla* | Tree | E | N | 148.86 | 77.48 |
| 143 | 1812 | *Abies nephrolepis* | Tree | E | N | 113.93 | 56.69 |
| 144 | 1812 | *Acer barbinerve* | Tree | D | B | 184.75 | 22.94 |
| 145 | 1812 | *Actaea asiatica* | Herb |  |  | 57.82 | 46.71 |
| 146 | 1812 | *Adenophora remotiflora* | Herb |  |  | 98.09 | 45.57 |
| 147 | 1812 | *Angelica gigas* | Herb |  |  | 127.6 | 33.09 |
| 148 | 1812 | *Aquilegia oxysepala* | Herb |  |  | 210.54 | 30.84 |
| 149 | 1812 | *Aruncus sylvester* | Herb |  |  | 157.5 | 19.53 |
| 150 | 1812 | *Athyrium melanolepis* | Herb |  |  | 82.03 | 49.74 |
| 151 | 1812 | *Betula ermanii* | Tree | D | B | 164.05 | 35.72 |
| 152 | 1812 | *Caltha palustris* | Herb |  |  | 84.31 | 46.69 |
| 153 | 1812 | *Carex forficula* | Herb |  |  | 101.01 | 39.74 |
| 154 | 1812 | *Carex pilosa* | Herb |  |  | 124.94 | 44.22 |
| 155 | 1812 | *Carex siderosticta* | Herb |  |  | 92.51 | 53.96 |
| 156 | 1812 | *Circaea cordata* | Herb |  |  | 79.75 | 29.47 |
| 157 | 1812 | *Clematis koreana* | Herb |  |  | 103.67 | 31.57 |
| 158 | 1812 | *Clintonia udensis* | Herb |  |  | 41.01 | 71.98 |
| 159 | 1812 | *Dryopteris crassirhizoma* | Herb |  |  | 71.77 | 54.47 |
| 160 | 1812 | *Dryopteris expansa* | Herb |  |  | 79.75 | 48.36 |
| 161 | 1812 | *Fragaria vesca* | Herb |  |  | 261.58 | 17.17 |
| 162 | 1812 | *Geranium wilfordii* | Herb |  |  | 135.57 | 48.52 |
| 163 | 1812 | *Lactuca sativa* | Herb |  |  | 123.61 | 29.65 |
| 164 | 1812 | *Larix gmelinii* | Tree | D | N | 95.7 | 58.96 |
| 165 | 1812 | *Lonicera caerulea var. edulis* | Shrub | D | B | 211.33 | 23.1 |
| 166 | 1812 | *Lonicera praeflorens* | Shrub | D | B | 247.22 | 21.23 |
| 167 | 1812 | *Matteuccia struthiopteris* | Herb |  |  | 58.48 | 44.43 |
| 168 | 1812 | *Parasenecio komarovianus* | Herb |  |  | 66.08 | 43.1 |
| 169 | 1812 | *Picea jezoensis* | Tree | E | N | 152.66 | 46.61 |
| 170 | 1812 | *Pinus koraiensis* | Tree | E | N | 101.01 | 51.91 |
| 171 | 1812 | *Poa alpina* | Herb |  |  | 154.18 | 37.65 |
| 172 | 1812 | *Polemonium caeruleum* | Herb |  |  | 123.61 | 25.45 |
| 173 | 1812 | *Polygonatum odoratum* | Herb |  |  | 53.17 | 77.49 |
| 174 | 1812 | *Rhododendron aureum* | Shrub | E | B | 311.02 | 22.72 |
| 175 | 1812 | *Ribes burejense* | Shrub | D | B | 112.22 | 24.19 |
| 176 | 1812 | *Saposhnikovia divaricata* | Herb |  |  | 291.65 | 25.37 |
| 177 | 1812 | *Solidago pacifica* | Herb |  |  | 131.59 | 29.97 |
| 178 | 1812 | *Sorbus pohuashanensis* | Tree | D | B | 122.81 | 36.25 |
| 179 | 1812 | *Trollius japonicus* | Herb |  |  | 74.43 | 49.67 |
| 180 | 2008 | *Aconitum monanthum* | Herb |  |  | 132.91 | 40.02 |
| 181 | 2008 | *Aquilegia oxysepala* | Herb |  |  | 313.68 | 29.21 |
| 182 | 2008 | *Bupleurum euphorbioides* | Herb |  |  | 188.21 | 16.8 |
| 183 | 2008 | *Carex siderosticta* | Herb |  |  | 187.41 | 48.55 |
| 184 | 2008 | *Halenia corniculata* | Herb |  |  | 221.3 | 24.35 |
| 185 | 2008 | *Pyrrosia petiolosa* | Herb |  |  | 197.78 | 35.75 |
| 186 | 2008 | *Rhododendron aureum* | Shrub | E | B | 334.94 | 26.13 |
| 187 | 2008 | *Saussurea alpina* | Herb |  |  | 275.13 | 27.65 |
| 188 | 2008 | *Saxifraga stolonifera* | Herb |  |  | 95.7 | 48.35 |
| 189 | 2008 | *Silene jenisseensis* | Herb |  |  | 133.98 | 38.79 |
| 190 | 2008 | *Solidago pacifica* | Herb |  |  | 151.52 | 32.12 |
| 191 | 2008 | *Vaccinium uliginosum* | Shrub | E | B | 257.85 | 28.46 |
| 192 | 2357 | *Chrysanthemum oreastrum* | Herb |  |  | 63.8 | 41.99 |
| 193 | 2357 | *Hedysarum vicioides* | Herb |  |  | 267.16 | 31.28 |
| 194 | 2357 | *Papaver radicatum var. pseudo-radicatum* | Herb |  |  | 77.47 | 40.4 |
| 195 | 2357 | *Pyrrosia petiolosa* | Herb |  |  | 255.2 | 29.6 |
| 196 | 2357 | *Rhodiola angusta* | Herb |  |  | 70.18 | 46.96 |
| 197 | 2357 | *Rhododendron aureum* | Shrub | E | B | 347.7 | 31.46 |
| 198 | 2357 | *Saussurea alpina* | Herb |  |  | 322.98 | 27.89 |
| 199 | 2357 | *Vaccinium uliginosum* | Shrub | E | B | 181.43 | 23.61 |
