# Supplementary material for: Elevation-Related Variation in Leaf Stomatal Traits as a Function of Plant Functional Type: Evidence from Changbai Mountain, China
Source: PLoS One. 2014 Dec 17;9(12):e115395. doi: 10.1371/journal.pone.0115395 (PMC4269444; doi:10.1371/journal.pone.0115395)
Supplement: S2 Table — Correlation matrix for environmental variables. MAT = mean annual temperature, MAP = mean annual precipitation, SWC = soil water content, and = CO2 partial pressure. Pearson coefficients in bold and with asterisks indicate the correlation is significant at P<0.01. (docx) [file pone.0115395.s004.docx]

**Table S2. Correlation matrix for environmental variables.** MAT = mean annual temperature, MAP = mean annual precipitation, SWC = soil water content, and $P_{\text{CO}_{2}}$= CO_2_ partial pressure. Pearson coefficients in bold and with asterisks indicate the correlation is significant at *P*<0.01.

|  | Altitude | MAT | MAP | SWC | $P_{\text{CO}_{2}}$ |
| --- | --- | --- | --- | --- | --- |
| Altitude | 1 | **-0.997**** | **0.997**** | -0.701 | **-1.000**** |
| MAT |  | 1 | **-0.997**** | 0.655 | **0.990**** |
| MAP |  |  | 1 | -0.661 | **-0.996**** |
| SWC |  |  |  | 1 | 0.709 |
