# Supplementary material for: Elevation-Related Variation in Leaf Stomatal Traits as a Function of Plant Functional Type: Evidence from Changbai Mountain, China
Source: PLoS One. 2014 Dec 17;9(12):e115395. doi: 10.1371/journal.pone.0115395 (PMC4269444; doi:10.1371/journal.pone.0115395)
Supplement: S3 Table — Number of sampled species among plant functional types (PFTs) at each site along altitude on the Changbai Mountain, China. The percentage of species number in total amount at each site is given between parentheses (%). Notably, most of evergreen trees are coniferous gymnosperms. (docx) [file pone.0115395.s005.docx]

**Table S3.** **Number of sampled species among plant functional types (PFTs) at each site along altitude on the Changbai Mountain, China.** The percentage of species number in total amount at each site is given between parentheses (%). Notably, most of evergreen trees are coniferous gymnosperms.

| Altitude (m) | Tree | |  | Shrub | |  | Herb |
| --- | --- | --- | --- | --- | --- | --- | --- |
|  | Deciduous | Evergreen |  | Deciduous | Evergreen |  |  |
| 540 | 13(28.26) | 2(4.35) |  | 11(23.91) | 0 |  | 20(43.48) |
| 753 | 15(20.83) | 1(1.39) |  | 12(16.67) | 0 |  | 44(61.11) |
| 1286 | 4(18.18) | 9(40.91) |  | 3(13.64) | 0 |  | 6(27.27) |
| 1812 | 4(10.53) | 4(10.53) |  | 3(7.89) | 1(2.63) |  | 26(68.42) |
| 2008 | 0 | 0 |  | 0 | 2(16.67) |  | 10(83.33) |
| 2357 | 0 | 0 |  | 0 | 2(25) |  | 6(75) |
